# Setting the right tone

**DOI:** 10.7554/eLife.55543

**Published:** 2020-02-10

**Authors:** Tanita Casci, Elizabeth Adams

**Affiliations:** University of GlasgowGlasgowUnited Kingdom

**Keywords:** research culture, academic careers, academic promotion, responsible metrics, University of Glasgow

## Abstract

Improving the research culture of an institution may lead to a fairer, more rewarding and successful environment, but how do you start making changes?

The University of Glasgow was founded more than 550 years ago and currently welcomes over 5000 researchers working in a wide range of subjects across the sciences and the humanities. Feedback suggests that our research culture is already good, but we think that it could be even better. As the Head of Research Policy (TC) and the Researcher Development Manager (EA), we have spent the past few years working to update research culture at Glasgow. Based on our experiences, our advice to anyone trying to change the culture of their institution is to be practical, consistent, and to aim for progress, not perfection. Start even if you cannot see the end. The project is big, slow, fragmented: and yes, it is a fantasy to imagine that a university has, or should have, a single culture.

The recent research culture survey by the Wellcome Trust has highlighted what many of us would not dispute: that the pursuit of a narrow definition of research excellence, and of excellence at any cost, has limited the research endeavour and had an adverse impact on the wellbeing of researchers as well as the quality and reliability of the research they undertake. It is not too late to fix this issue, but solutions will emerge only once research organisations, funders, publishers and government coordinate their efforts to identify practical actions that can be implemented consistently across the research community.

Meanwhile, the complexity of the problem should in no way stop us from implementing changes within our own institutions. At Glasgow, we focus on fostering a positive research culture. To do so, we develop policies, guidance, communications, training and related initiatives that support the success of researchers at all stages of their career.

With the support of our senior management, we have introduced several initiatives that we hope will make our institution an inspiring place in which to develop a career — whether it is academic or administrative, operational or technical, or indeed something different altogether. Some of these initiatives are summarised in this post; in this article we will also share the lessons we learned along the way that might be useful to others.

## Start from what you know

Research culture is a hazy concept, which includes the way we evaluate, support and reward quality in research, how we recognise varied contributions to a research activity, and the way we support different career paths.

Of all the things you could do to improve research culture, start from the priorities that you think matter most to your organisation; those that reflect its values, fit with what your community really cares about, or align to the activities that are already in progress. If you can, line up your agenda to an external driver. In our situation, two prominent drivers are the UK Research Excellence Framework (an exercise that assesses the quality of research, including the research environment, at all UK universities), and the Athena Swan awards (which evaluate gender equality at institutional and local levels). Our research culture initiatives also work alongside everyday drivers from research funders and other bodies, such as concordats on research integrity, career development and open research data.

Even better, align your initiative to more than one agenda. For example, we are supporting transparency, fairness, accountability (and therefore quality, career development, and collaboration) by requesting that research articles deposited in our institutional repository follow the CRediT taxonomy, whereby the roles and responsibilities of each authors are laid down explicitly.

Once you know what you mean by culture, write it down and let people know. This will aid communication, keep everyone focussed, and avoid the misunderstanding that culture is a solution to all our problems (“The car parking is a nightmare. I thought we had a culture agenda!”).

At Glasgow we define a positive research culture as one in which colleagues (i) are valued for their contributions to a research activity, (ii) support each other to succeed, and (iii) are supported to produce research that meets the highest standards of academic rigour. We have then aligned our activities to meet these aims, for example by redesigning our promotion criteria to include collegiality, and creating a new career track for research scientists (see Box 1).

Box 1. Changing promotion criteria and career trajectories to foster a different research culture

At the University of Glasgow, academic promotion criteria are based on a 'preponderance approach': candidates need only meet the necessary criteria in four of the seven dimensions used to assess staff for promotion (academic outputs; grant capture; supervision; esteem; learning and teaching practice; impact; leadership, management and engagement). For the 2019–2020 promotions round, the University has also introduced a requirement to evidence collegiality as well as excellence in each of the four qualifying dimensions. The criteria recognise not only the achievement of the individual but also how that individual has supported the careers of others.

From 2019–2020 onwards, promotion criteria for the academic track also explicitly state that one of the four qualifying criteria should be either academic outputs or impact. By ‘impact’ we mean the evidenced benefits to society that have resulted from the research – these could be economic, societal, cultural, or related to health and policy. The new criteria therefore formally acknowledge that societal impact holds as much value to the institution as outputs, and that generating and evidencing impact takes time. It also ensures that staff does not feel under pressure to ‘do everything’. We will be monitoring the effect of these changes in mid 2020.

In addition, Glasgow has recently introduced a career pathway for research scientists: this track recognises and rewards the contributions made by researchers who have specialist knowledge and skills, such as bioinformaticians. The contributions and intellectual leadership provided by these roles are often not reflected in the traditional promotion criteria, which depend on lead or senior authorships. Research scientists can instead progress in their careers by demonstrating specialist work stream, as well as team contributions.

## Practice, not policy

Success will not come from issuing policies, but by making practical changes that signal “the way we do things around here”. Even if university policies are read, they will be forgotten unless the principles are embedded in standard practice. And if we are not serious about our practices, then we are not credible about our intentions.

Over 1500 organisations have signed DORA and have committed not to use unreliable proxies such as journal impact factors in research evaluation. Yet, even purging references to journal impact factors from all paperwork is no guarantee that these or other metrics will not be used. If we are serious about fair evaluation mechanisms, then we need to provide evaluation panels with meaningful information. At Glasgow, we ask applicants to describe in 100 words the importance of their output, and their contribution to it. Many organisations have switched to the use of narrative formats, for instance the Royal Society, or the Dutch research council (NWO). To show that we value all dimensions of research, we also ask for a commitment to open research and give parity of credit to academic outputs (such as papers) and the societal impact they create (see Box 1).

To ensure that changes are felt on the ground, we are embedding these priorities in annual appraisals, promotion and recruitment, so that the same expectations are encountered in every relevant setting. We have also included the importance of responsible metrics in recruitment training, and will be working with our colleagues in human resources to ensure that local conversations with hiring managers are consistent with our metrics policy (see Box 2).

Box 2. Responsible metrics

The policy on the responsible use of metrics means ensuring that the mechanisms we use to evaluate research quality are appropriate and fairly applied. For example, we need to make sure that quantitative indicators are suitably benchmarked and normalised by subject, and that they are used along qualitative ones. This is to avoid the over-reliance on single-point metrics (such as research funding) and over-use of unreliable proxies for quality (such as journal impact factors).

The policy describes our approach to evaluating the quality of our outputs, our supervision and our grant capture. The proof, however, is in the way the policy is implemented in practice. For example, applicants to our strategic recruitment schemes are requested to select their four best outputs, describe the significance of each output to the field (without relying on impact factors), and narrate their contribution to the work. Applicants are also asked to describe their commitment to open research. This approach allows the recruitment panel to obtain a more rounded impression of the candidate and, we hope, reduces the use of unhelpful proxies such as length of publication list or journal impact factors.

## Start, even if you cannot see the finish line

Once you have decided on the general direction, start by doing something without worrying about scoping the project from start to finish.

At Glasgow we started by doing a 360-degree review of our provision for research integrity: this was not just about the training but also about raising the visibility of this agenda in the community. We did not call it ‘culture’ then, but we realised that progress would come from communicating the dimensions of good practice (e.g. open research) rather than by sanctioning breaches of conduct. That exercise gave us experience of getting support from senior management, managing a cross-institutional working group, and getting buy-in from the academic body through the establishment of a network of 29 integrity advisers. These individuals champion this agenda to researchers, contribute to training and policy and also participate in research misconduct panels.

From integrity, we moved to open research, and from there, to careers. It started with compliance, and progressed towards culture. Do not wait for the rules to come to you. Make your own. Have confidence that once projects are initiated, they will suggest future courses of action.

## Shout about it

If you want to be noticed, it helps to over-communicate. If your project serves more than one agenda, then your colleagues in, say, human resources, the library, the research office, and the equality, diversity, and inclusion team will already be helping you to amplify the message. We have set up a Culture and Careers group that brings together a range of relevant professional groups and colleagues. Focusing on our culture activities and the training that we can provide to staff and students helps us to share knowledge and to highlight where different agendas can reinforce each other.

Make the framework easy to understand: at Glasgow we talk about supporting what we value (e.g. CRediT), recognising what we value (e.g. our promotion criteria), and celebrating those values, for instance with our recently launched research culture awards. These highlight outstanding activities that promote collegial behaviours and contribute to a positive research culture. In 2019, over 30 applications were received from across the institution, reflecting a variety of career stages, coming from academic, technical and professional services roles, and ranging from groups of researchers to individual staff. The awards have changed the conversation as to what culture actually is.

But equally do not fret if colleagues do not know how your various activities fit together under a ‘culture’ agenda. It is far more important that researchers embrace the activities themselves (see “Practice, not policy” above).

Communication takes legwork, so use any channel you have. Present at committees, consult with different disciplines and career stages. Speak to the willing. Welcome the challenge. Bring together different voices in a discussion forum. For example, we recently organised a research culture event involving action-oriented conversations with academics, administrators, funders, societies, and publishers; this helped to build our evidence base, share perspectives and move forward institutional thinking in relation to key areas of culture (see the illustration for a summary of the discussion).

**Figure 1:**
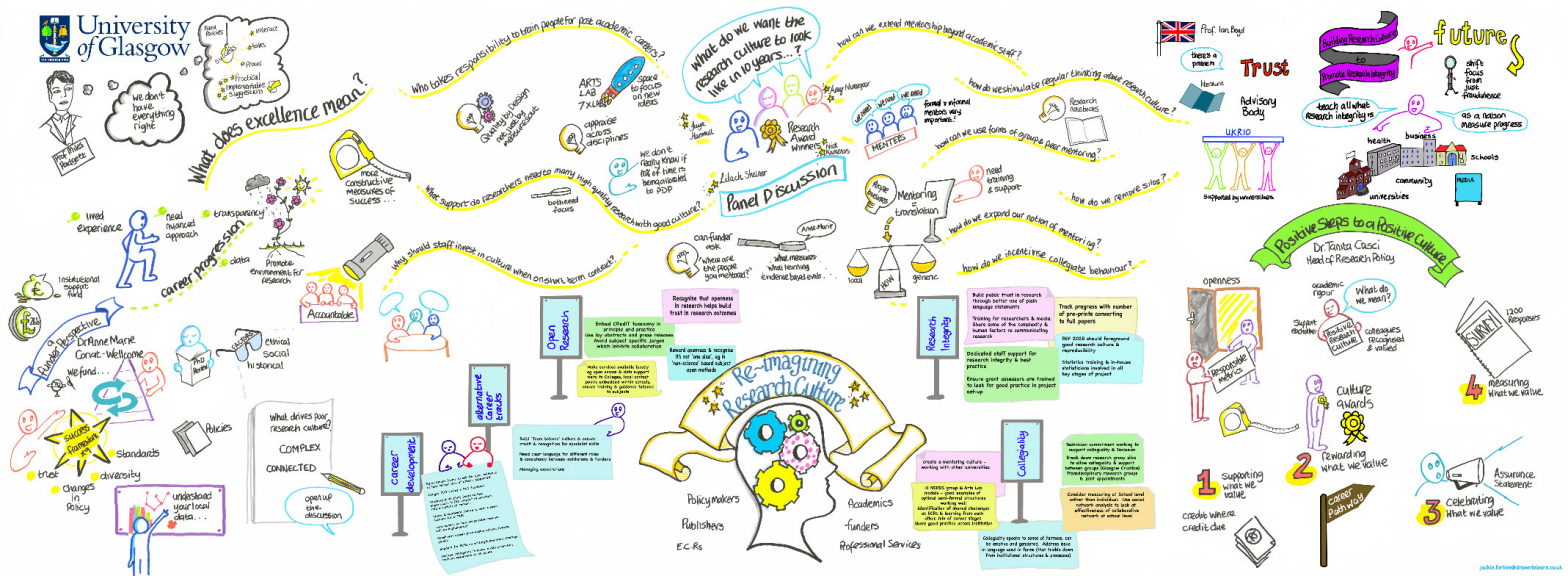
Map of the ideas discussed at the Re-imagining research culture workshop organised at the University of Glasgow in September 2019.

Jacquie Forbes at drawntolearn.co.uk (CC BY 4.0)

A research culture survey allowed us to assess how we were doing. It gathered examples of good practice (for example, that the community appreciated reading groups and the opportunity for internal peer review) and it highlighted the aspects of research our staff were comfortable with (open access, for instance). It also pointed us towards what people wanted to know more about, such as how to increase the visibility of their research. Together, the event and survey have informed our next actions (you can access the question set here) and our action plan for the next five years.

## No such thing as a single culture

If you work in a research organisation, you are probably relaxed about the fact that different parts of the institution have their own priorities, as befits the disciplinary community.

Institution-wide projects should be designed to address the broad ambitions of the university: for example, all areas of the university can participate in the research culture awards or meet the requirement for collegiality in our promotion criteria.

Each discipline can then be invited to implement the culture programme that suits them. Getting this right requires a bit of flexibility, some confidence that things will not unravel, but also clear leadership. Some institutional glue can be provided by sharing case studies between areas, which is helped by collecting feedback on how policies and guidance are being implemented at the university level. For example, our new guidance on embedding equality, diversity and inclusion in conferences and events contains a weblink to a feedback survey. We hope that this will help us to pinpoint where colleagues are struggling to implement best practice, perhaps due to other organisational challenges such as funding, lack of clear guidance or procurement.

## What’s next?

We have published an action plan for our 2020–2025 university strategy, which covers career development, research evaluation, collegiality, open research and research integrity. The starting point will be to focus on supporting career development, on helping researchers to enhance their visibility, and on developing an informed and committed leadership across the university.

We have also published an institutional statement to highlight the road travelled and our future plans. All the while, we are drawing inspiration from others: the Wellcome Trust and the Royal Society, and the progressive policies introduced by publishers such as PLoS, eLife, Wiley, and F1000. We are excited by the launch of initiatives that will inform better decision-making in the culture space, and online groups for sharing ideas. We want to be a part of organisations, such as the UK Reproducibility Network, that identify priorities and work together in implementing them.

We are also casting our eyes towards broader aspects of culture: how do we define and encourage research creativity, how do we make more time, and how might we extend the scope of our actions beyond research staff to all those that contribute to research?

Culture does not happen at the expense of excellence; an updated culture is what will allow even more of us to excel.

